# Current Treatment Paradigm and Approach to Advanced Hepatocellular Carcinoma

**DOI:** 10.7759/cureus.75471

**Published:** 2024-12-10

**Authors:** John Patresan, Harsh Patel, Karthik Chandrasekaran, Griffin Reynolds

**Affiliations:** 1 Hematology and Oncology, Roger Williams Medical Center, Boston University School of Medicine, Providence, USA; 2 Gastroenterology and Hepatology, New York-Presbyterian Brooklyn Methodist Hospital, Weill Cornell Medicine, Brooklyn, USA; 3 Internal Medicine and Gastroenterology, New York-Presbyterian Brooklyn Methodist Hospital, Weill Cornell Medicine, Brooklyn, USA

**Keywords:** hepatocellular carcinoma (hcc), liver transplant, transarterial chemoembolization (tace), tyrosine kinase inhibitors (tki), unresectable hcc

## Abstract

Hepatocellular carcinoma (HCC) is one of the most common forms of primary liver cancer worldwide. Herein, we present a review article that provides a broad overview of the current landscape of HCC, including the etiology, potential risk factors, and molecular pathways that can serve as potential therapeutic targets. The risk factors tend to vary depending on the geographic distribution; hepatitis B-induced cirrhosis and HCC occur more frequently in Asia and Sub-Saharan Africa, whereas metabolic disorders are the culprits in Western Europe and the Americas. The exact molecular alterations that drive hepatocarcinogenesis have yet to be elucidated; however, a complex interplay exists between oxidative stress and chronic inflammation. Diagnostic modalities such as tri-phasic MRI or CT also have distinct patterns for HCC, which aid significantly in diagnosis. Furthermore, the review aims to highlight treatment strategies, including transplantation, locoregional radiation therapies, and interventional radiological techniques such as chemotherapy or radioembolization. Finally, systemic therapies will be discussed, taking advantage of molecular pathways that influence cellular proliferation and survival as well as immunotherapy.

## Introduction and background

Primarily, liver cancer is the sixth most common cancer worldwide, with approximately 906,000 new cases and the third most common cause of cancer mortality worldwide, with roughly 830,000 deaths per year [1]. Hepatocellular carcinoma (HCC) is the main type of primary liver cancer, accounting for 75-85% of cases [1]. The main risk factor for HCC is cirrhosis due to the hepatitis B virus or hepatitis C virus, which accounts for nearly 80-90% of cases [2]. Additional risk factors include aflatoxin-contaminated food, alcohol use, and metabolic syndrome-related sequelae such as elevated BMI and type 2 diabetes mellitus. Though a global issue, the HCC disease burden varies geographically; for example, HCC distribution remains highest in areas with endemic HBV infection, such as East and South Asia and sub-Saharan Africa [2]. In the Western Hemisphere and Europe, cases are largely due to HCV and the rising prevalence of metabolic disorders such as non-alcoholic fatty liver disease [2].

Despite improvements in screening and diagnosis of HCC, the advanced stage remains the most common presentation at diagnosis [3]. The BRIDGE study highlighted that over 50% of patients diagnosed with HCC were in advanced or terminal stages, with even higher proportions in limited-resource countries [4]. A considerable number of advanced-stage HCC patients are unfortunately unsuitable for liver resection surgery or local treatment, thus limiting many management options. The prognosis for advanced-stage HCC is extremely poor, with the one-, two-, and three-year survival rates at 29%, 16%, and 8%, respectively, and a median overall survival of less than six months [5].

## Review

Treatment paradigm

Currently, the treatment of HCC is primarily based on a multifactorial approach. Of all the staging systems available worldwide, the Barcelona Clinic Liver Cancer (BCLC) is the most widely used prognostic staging system for HCC [6-7]. It incorporates tumor burden, liver reserve, and performance status to classify HCC into stages (Table 1), which are used to identify optimal treatment modalities.

**Table 1:** Stages of HCC (2022) HCC: hepatocellular carcinoma, ECOG: Eastern Cooperative Oncology Group Table Credit: John Patresan M.D.

Stage	Criteria for staging
Stage 0	Very early-stage HCC, single lesion less than ≤2 cm with preserved liver function, ECOG performance status 0
Stage A	Early stage, single, or ≤3 nodules each ≤3 cm in size with preserved liver function, ECOG performance status 0
Stage B	Intermediate stage, multinodular, preserved liver function, ECOG performance status 0
Stage C	Advanced stage, portal invasion, extrahepatic spread, preserved liver function, ECOG performance status 1-2
Stage D	Terminal stage, any tumor burden, end stage liver function, ECOG performance status 3-4

Curative treatments such as surgical resection, tumor ablation, and liver transplantation are generally reserved for very early (stage 0) and early stage (stage A) HCC. Unfortunately, due to poor surveillance programs, a vast majority of patients will present with BCLC stage C or D at the time of diagnosis [3]. According to BCLC, the presence of multi-nodular disease, portal vein invasion, and performance status 1 or 2 is enough to classify one as having intermediate or advanced disease where palliative care would be indicated [5]. Additionally, the presence of portal hypertension would be a contraindication to liver resection as a treatment alternative, in which case liver transplant or ablation techniques could be employed in those scenarios [5]. While the BCLC classification was previously used to provide strict boundaries and limitations to therapies, current advances in medicine have since blurred such boundaries. Certain interventional or ablative therapies can potentially help bridge a patient with an initial large tumor burden to possible resection. Patients with advanced HCC and poor liver function may now be able to tolerate liver resection or be given consideration for systemic therapies. As such, there has been a greater emphasis on the development of non-surgical treatment options such as locoregional therapy and systemic treatment with multi-kinase inhibitors and immunotherapy.

Liver Transplant

Liver transplantation is one of very few curative treatment techniques. The current, widely accepted eligibility criteria for transplantation is outlined by the Milan Criteria: a single lesion smaller than 5 cm or two to three lesions, each less than 3 cm, and no major vessel or extrahepatic involvement [8]. It is largely considered the benchmark for consideration of liver transplantation due to its remarkable five-year survival rate of 75% [9]. The Milan Criteria (Table 2) has received some criticism due to its extremely stringent criteria, potentially excluding a significant number of patients who may benefit from liver transplants. Only 6% of patients with HCC are eligible for liver transplants per the Milan Criteria [10]. Several attempts have been made to extend the Milan Criteria to include a broader group of patients while maintaining its high survival rate. A group from the University of California San Francisco (UCSF) created an extended selection criteria (Table 2) to include one tumor less than 6.5 cm or two to three tumors, each less than 4.5 cm with a total length less than 8 cm [11]. Patients within the UCSF criteria had a one- and five-year survival rate of 90% and 75%, respectively [12]. A large-scale study that included patients beyond the Milan Criteria but within the USCF Criteria demonstrated non-inferiority of the UCSF Criteria to the Milan Criteria using one-, two-, three-, and four-year survival rates as primary endpoints [13]. In 2009, the Milan group attempted to modify the criteria further by extending the criteria to up to seven tumors with the sum size of the largest tumor, 7 cm. The so-called “Up-To-Seven” criteria without microvascular invasion had a five-year overall survival rate of 71.2% [14]. Despite multiple studies analyzing and supporting the effectiveness of the up-to-seven criteria, it has yet to be as widely accepted as the Milan Criteria. The scarcity of the liver allograft donor pool and the stringent Milan Criteria are major limitations of liver transplantation and leave many HCC patients to consider alternative treatments. Indeed, liver transplantation only offers a moderate survival benefit for patients with Child-Pugh Class A cirrhosis and early-stage HCC when compared to liver resection or ablative treatments [15]. This may suggest that liver transplants for early-stage HCC and compensated cirrhotic patients may not make sufficient use of the liver transplant pool, and such patients can be adequately treated with liver resection or ablation instead [16].

**Table 2:** Transplant criterion UNOS-DS: United Network Organ Sharing Downstaging, UCSF: University of California San Francisco Table Credits: John Patresan M.D., Harsh Patel M.D.

Milan criteria	UNOS-DS criteria	UCSF criteria
Single tumor <5 cm	One tumor 5.1-8 cm	One tumor ≤6.5 cm
Two to three tumors <3 cm	Two or three tumors, each ≤5 cm. Four to five tumors ≤3 cm with the sum of the longest diameter of each tumor ≤8	Two to three tumors, each ≤4.5 cm, with the sum of the longest diameter of each tumor ≤8 cm
No imaging evidence of vascular invasion	No imaging evidence of vascular invasion	No imaging evidence of vascular invasion
No imaging evidence of extrahepatic metastatic disease	No imaging evidence of extrahepatic metastatic disease	No imaging evidence of extrahepatic metastatic disease

Owing to the success of the UCSF criteria and its excellent post-liver transplant survival with low rates of recurrence, the United Network Organ Sharing (UNOS) adapted its system in 2017 and created a uniform downstaging criterion (Table 2) known as UNOS-DS (downstaging). This criterion was defined as patients with HCC and one tumor with a size greater than or equal to 5 cm; two to three tumors, each less than or equal to 5 cm in size; or four to five tumors, each less than or equal to 3 cm in size, with the total sum of all lesions being less than 8 cm in size [16-68]. The principal goal is to determine which patients have the potential to be eligible for liver transplants. Downstaging via locoregional therapies remains a bridge to liver transplant.

For downstaging, typically, patients require good synthetic liver function (Child-Pugh A or B) with a bilirubin of less than or equal to 3 mg/dl. Transarterial chemoembolization (TACE) is the most commonly used strategy, although Yttrium-90 (Y90) has shown efficacy but requires more data [16-68]. While there is no specific guidance on observation time, a period of at least three months after downstaging successfully is recommended prior to live donor liver transplantation [16-68]. The choice of therapeutic intervention for downstaging, as well as the period of observation after treatment to determine eligibility for transplant, varies by institution. Given the heterogeneity of approaches to downstaging, more research is needed to help standardize downstaging approaches for liver transplants in HCC.

Liver Resection

Liver resection is another effective curative treatment technique. It involves the total removal of a liver segment fed by a branch of the portal vein and hepatic artery [16]. Resection remains one of the more effective treatment modalities, with a five-year survival rate of 50-70% [17]. En-bloc resection of the tumor and its entire portal vein territory is recommended, as HCC spread occurs primarily through the portal vein [18]. The success rate of liver resection remains largely dependent on liver function (MELD score, Child-Pugh Class, and ECOG status). In general, only patients with Child-Pugh Class A cirrhosis or those with MELD scores less than 10 can undergo resection safely [17]. Evaluation of future liver remnant volume (FLRV %) is also an important test for patients undergoing liver resection. To avoid postoperative liver failure, the target FLRV % is 40% for patients with chronic liver disease and 30% for those without chronic liver disease [19]. Per BCLC staging, surgical resection is only offered as a curative option for stage 0 or A HCC patients, while other stages are defined as non-operable [20-21]. Given modern advances in perioperative management and surgical techniques, the indication for liver resection can partially be expanded to more advanced stages [22-23]. A 2015 multicenter study comparing liver resection across different BCLC stages showed that patients in stages B and C may also benefit from resection over locoregional therapies [24]. Another randomized controlled trial demonstrated that liver resection significantly increased overall survival in HCC patients outside the Milan Criteria compared to locoregional therapy (trans-arterial chemoembolization) [25]. Nonetheless, the reality remains that nearly half of HCC patients at the time of diagnosis will not be candidates for transplant or resection. There is a large need for less invasive treatment options, such as locoregional and systemic treatments.

Locoregional Therapy

Interventional or locoregional therapies can also treat advanced HCC with a wide range of curative and palliative intents. They can be used in combination with resection, bridging the waiting time to transplantation or downstage advanced tumors to fulfill the criteria of resection or transplantation [16]. Additionally, patients with advanced-stage HCC with low-grade extrahepatic involvement (BCLC stage C) may also benefit from locoregional therapy to help slow tumor progression, which is ultimately the leading cause of their death [26]. There is a broad spectrum of locoregional therapies, such as TACE, transarterial radioembolization (TARE) with Y90, radiofrequency ablation (RFA), and microwave ablation (MWA), among many others [26].

RFA is the most widely used ablative technique for advanced HCC. It relies on the principle of ion agitation and heat generated, otherwise known as the Joule Effect [27]. It works most effectively in small, centrally situated tumors less than 2 cm in size. It is an appropriate treatment strategy for BCLC stage 0 and A patients who are not eligible for surgical resection [16]. RFA carries a local sustained complete response rate of 97.2% after a follow-up of 31 months [28]. In a cohort study carried out primarily in Child-Pugh Class A cirrhosis patients with very early-stage HCC tumor size less than 2 cm, RFA provided good local control with a local recurrence rate of just 5% [29]. However, the efficacy of RFA diminishes with increasing tumor size [27]. Another important consideration is that tumors proximal to any large vessels may impair RFA efficacy due to the cooling effect of proximal blood flow, otherwise known as the heat sink effect [30].

TACE is an alternative form of locoregional therapy currently recommended for BCLC stage B (intermediate) HCC patients [31]. TACE targets the arterial hypervascularization of HCC via embolization of the tumor blood supply with a chemotherapeutic agent such as doxorubicin or cisplatin [16]. In patients satisfying the criteria for liver resection, TACE resulted in an inferior overall survival compared with liver resection [25]. However, several randomized controlled trials have shown improved overall survival with TACE compared to supportive care in BCLC stage B patients beyond liver resection criteria [32-33]. Since TACE can compromise hepatic blood flow and cause acute deterioration of liver function, liver function must be carefully evaluated in the preoperative period [34]. While TACE is generally indicated for BCLC stage B patients with non-resectable disease, careful consideration must be taken into patient selection as many stage B patients may have underlying cirrhosis and poor hepatic reserve. The risk for acute liver failure significantly increases in HCC patients with Child-Pugh Class B cirrhosis, while TACE is contraindicated in patients with Child-Pugh Class C cirrhosis [35]. Additionally, TACE is not indicated in HCC patients with evidence of portal vein tumor thrombosis (a major complication of HCC), as embolization during TACE can further compromise hepatic blood flow and contribute to adverse outcomes [34].

TARE is another widely used interventional treatment for managing HCC. Y90 is a radiation-emitting isotope introduced via a microcatheter into the hepatic artery branch feeding the target tumor, allowing for ischemic necrosis of the tumor site [36]. One of the primary benefits of TARE is that it can be safely performed across BCLC stages A-C and selectively in some BCLC stage D patients [37]. Additionally, due to a less dominant effect on hepatic artery blood flow than TACE, TARE can be safely performed in patients with portal vein thrombosis or tumor thrombosis [38]. Less promising, two recent phase III trials in 2017 and 2018 failed to demonstrate the benefit of TARE over sorafenib, a widely accepted systemic therapy for HCC. The trials compared TARE to sorafenib in locally advanced, inoperable HCC and reported no significant difference in overall progression-free survival [39-40]. Interestingly, however, TARE did demonstrate a lower incidence of hepatic tumor progression and had a significantly more favorable toxicity profile when compared to sorafenib [39-40].

Systemic Therapy

Systemic therapies for advanced HCC have experienced a considerable amount of advancement in research in the past decade. Hepatocarcinogenesis is associated with alterations in the molecular pathways, which lead to the abnormal growth of hepatocytes [41-69]. There has been a concentrated effort to identify these molecular pathways to narrow down the targets of systemic chemotherapeutic drugs, one of which is the kinase pathway. In HCC, tumor activity's growth and continued activation are affected by many growth factors (GF) (i.e., vascular endothelial GF, hepatocyte GF, epidermal GF, fibroblast GF, and platelet-derived GF). Each of these GF is a receptor signal in the tyrosine kinase pathway, which, when activated, induces gene transcription that promotes cell proliferation [41-69]. The study of agents that inhibit these GF, tumor proliferation, has been a breakthrough in treating advanced HCC. Unfortunately, due to the underlying chronic liver disease (cirrhosis) that often accompanies HCC, the applicability of most chemotherapeutic regimens has been severely limited. Patients often tolerate these agents poorly, and many will self-discontinue treatment prior to achieving any clinical benefit. To date, there has only been a handful of systemic chemotherapy regimens with proven benefits in HCC, which will be discussed in detail in this review article.

Sorafenib is an oral multi-kinase inhibitor with activity that can inhibit tumor cell proliferation by blocking the activity of the kinases in the RAS/RAF/MEK/ERK signaling pathway. In addition, it functions to block angiogenesis through the targeting of vascular endothelial GF (VEGF)-2,3 PDGFR-β, hepatocyte factor receptor (c-Kit), and Fms-like tyrosine kinase (FLT-3) [42]. In 2007, the FDA approved sorafenib as a first-line treatment for advanced HCC after the results of the Sorafenib Hepatocellular Carcinoma Assessment Randomized Protocol (SHARP) study were published. The SHARP study was a phase III randomized, controlled clinical trial that compared sorafenib to a placebo. The study demonstrated that patients with advanced HCC had a 2.8-month median overall survival benefit for sorafenib (10.7 months vs. 7.9 months) [43]. A second phase III trial was carried out in Asia with a different cohort of patients and again confirmed that sorafenib improved median overall survival compared to placebo [44]. Both trials were carried out in patients with preserved liver function (Child-Pugh Class A cirrhosis) and good functional status (ECOG 0-1) but were not amenable to surgery or locoregional therapies [43-44]. Sorafenib was also found to have a narrow therapeutic window. Despite improvement in overall survival, many patients discontinue treatment due to poor tolerance of severe adverse effects such as diarrhea, weight loss, and palmar-plantar erythrodysesthesia (hand-foot skin reaction) [43-44]. Several reports showed that early adverse reactions, particularly hand-foot skin reactions, were associated with a longer median overall survival compared to those who did not develop any adverse reactions (18.2 months vs. 10.1 months) [45]. Considering the SHARP trial, sorafenib has become the mainstay of palliative treatment in BCLC stage C patients [46].

The GIDEON trial helped highlight the use of sorafenib in Child-Pugh Class A versus Class B cirrhosis patients. The trial confirmed that median overall survival was shorter in Class B patients (5.5 months) compared to Class A patients (13.6 months) [47]. However, time to progression and incidence of adverse events were similar across both subgroups, though the severity of adverse events was greater in Class B patients [48]. Hence, sorafenib is still an option for Child-Pugh B patients with HCC. Eventually, 60-70% of HCC patients who start treatment will progress on sorafenib [49]. Despite achieving approval as a first-line treatment for advanced, non-resectable HCC, sorafenib has a marginal efficacy and toxicity profile, which has prompted research for the development of other agents.

In 2018, the FDA approved lenvatinib as an alternate first-line treatment option for patients with advanced HCC. Lenvatinib is another broad target multi-kinase inhibitor with activity against VEGF, fibroblast GF receptors, and PDGFR. The REFLECT trial tested the safety and efficacy of lenvatinib to sorafenib in patients with unresectable HCC. The study confirmed the non-inferiority of lenvatinib to sorafenib when measuring overall survival [50]. However, the study also demonstrated statistically significant higher objective response rates, progression-free survival, and time to progression in the lenvatinib arm versus the sorafenib arm [50]. In 2020, an analysis of a subset of patients from Japan confirmed the same findings from the REFLECT trial [51]. Although many secondary endpoints of the REFLECT trial reported positive findings, lenvatinib also reported significantly higher rates of high-grade adverse events than sorafenib [50]. Up to 37% of patients in the REFLECT trial required a lenvatinib dose reduction, and up to 9% withdrew entirely due to treatment-related adverse events [45,50]. Lenvatinib has a very similar adverse effect profile to sorafenib, with diarrhea, fatigue, anorexia, and palmar-plantar erythrodysesthesia being the most reported (50). It is additionally associated with higher rates of proteinuria and dysphonia [52]. Lenvatinib is the first agent to show equal or better results than sorafenib in the treatment of advanced HCC and has become a breakthrough for the study of additional multi-kinase inhibitors.

Over the past several years, many oncological breakthroughs have been made in cancer immunotherapy. While primarily studied and approved for hematological and other solid organ tumors, the study of immunotherapy regimens for HCC has rapidly emerged. Immune checkpoint inhibitors targeting the programmed cell death protein-1/programmed death ligand-1 (PD-1/PD-L1) pathway and the cytotoxic T-lymphocyte-associated antigen 4 (CTLA-4) pathway are the two most studied immunotherapy mechanisms in HCC [53]. PD-L1 is expressed on HCC tumor cells and interacts with PD-1 on host-activated T cells, thereby leading to the inactivation of T cells [53-54]. This blockade suppresses the host immune response to HCC tumor cells and attenuates T cell activity. The CTLA-4 receptor is an immunoglobin-related receptor that serves a role in downregulating host T cell response, providing another novel mechanism for which HCC tumor cells can become susceptible to immune-mediated destruction [54-55].

Nivolumab, a monoclonal antibody inhibiting the PD-1 receptor, was one of the first widely tested immune checkpoint inhibitors. The CheckMate 040 study demonstrated a 72% overall survival rate at 6 months with a favorable response rate of 20% of nivolumab in treating advanced HCC in patients who had progressed on sorafenib [56]. A subsequent expansion study tested nivolumab on HBV- and HCV-related HCC patients with similar overall survival rates (82.5% and 70.8%, respectively) [57]. Following its approval as a second-line agent for the treatment of advanced HCC, nivolumab was evaluated in the CheckMate 459 trial as a first-line agent for advanced HCC compared to sorafenib. In this head-to-head trial, nivolumab had a similar response rate and improvement in overall survival compared to sorafenib as a first-line agent. However, the primary endpoint of overall survival did not reach statistical significance [58]. Nivolumab's overall adverse effect profile is also favorable, with an overall incidence rate of less than 5% [56]. The most reported adverse effects were immune-mediated events such as autoimmune hepatitis, colitis, pneumonitis, and uveitis.

Pembrolizumab is another anti-PD-1 monoclonal antibody being tested for use in advanced HCC therapy. The Keynote-224 trial tested the efficacy and safety of pembrolizumab in advanced HCC patients with disease progression on sorafenib. The primary endpoint studied was the overall response rate. In the study, 44% had stable disease, and 17% achieved the objective response rate, including one complete response rate [59]. This result was also consistently seen in the follow-up Keynote-240 study. However, the primary endpoints of overall and progression-free survival were unmet [60]. Dual immunotherapy approaches were also examined in the CheckMate 040 study, which tested the combination of nivolumab (PD-1 inhibitor) and ipilimumab (CTLA-4 inhibitor), which showed a median overall survival of 23 months [61].

More recently, two landmark approvals in systemic therapy approaches have come from two major trials, the IMBRAVE-150 study and the HIMALAYA trial. The former looked at the combination of atezolizumab (PD-L1 inhibitor) and bevacizumab, a monoclonal antibody that inhibits the action of VEGF. This approach combined immunotherapy with alterations of the tumor microenvironment and angiogenesis. In patients with unresectable HCC, atezolizumab, combined with bevacizumab, improved overall survival and progression-free survival compared to sorafenib [62]. Overall survival at 12 months was 67.2% (95% CI, 61.3 to 73.1) with atezolizumab-bevacizumab and 54.6% (95% CI, 45.2 to 64.0) with sorafenib. Median progression-free survival was 6.8 months (95% CI, 5.7 to 8.3) and 4.3 months (95% CI, 4.0 to 5.6) in the respective groups (hazard ratio for disease progression or death, 0.59; 95% CI, 0.47 to 0.76; p<0.001) [62].

The HIMALAYA trial was a three-arm study that compared the combination of tremelimumab (CTLA-4 inhibitor) with durvalumab (PD-L1 inhibitor), durvalumab monotherapy, and sorafenib in patients with unresectable HCC. Overall survival at 36 months was 30.7%, 24.7%, and 20.2%, respectively (63). Durvalumab alone was also deemed to be non-inferior to sorafenib [63]. The tremelimumab-durvalumab approach is a useful alternative when patients may have a contraindication to bevacizumab due to high bleeding risk or poor wound healing.

Unfortunately, once patients with unresectable disease either progress on sorafenib or another systemic therapy, the options become limited. However, a few options are listed in the National Comprehensive Cancer Network guidelines that can be used in the second line. Cabozantinib is a multi-kinase inhibitor approved for patients with previously treated advanced HCC. Treatment with cabozantinib resulted in longer overall and progression-free survival than placebo (10.2 vs. 8.0 months with placebo) [64]. However, the adverse effect incidence demonstrated that 68% of patients in the treatment arm suffered from grade 3-4 toxicities, including palmoplantar erythrodysesthesia, hypertension, and transaminitis. Regorafenib, another multi-kinase inhibitor, improved overall survival with a hazard ratio of 0.63 (95% CI 0.50-0.79; one-sided p<0·0001); median survival was 10·6 months (95% CI 9·1-12·1) for regorafenib versus 7·8 months (6·3-8·8) for placebo [65]. One criticism of the studies is that sorafenib was used as the control arm, which was the only approved systemic option at the time. However, we currently have more efficacious modalities. Therefore, if a patient progresses on an immunotherapy combination, further trials are needed to deduce the appropriate second-line agent.

Lastly, the current landscape of systemic therapies for advanced HCC is a dynamic and progressing field. The most recent addition to the first-line treatment options in advanced HCC is tislelizumab (PD-1 inhibitor), evaluated in the phase III RATIONALE-301 study against sorafenib [66]. With a 36-month follow-up, tislelizumab was not inferior to sorafenib. There has been interest in exploring the role of bispecific antibodies that simultaneously inhibit PD-1 and CTLA-4, which are currently undergoing phase I/II trials [66-67].

## Conclusions

HCC remains one of the more prevalent cancers that occur globally. Screening efforts and improved imaging modalities using triple-phase MRI or CT have been instrumental in diagnosing and managing these tumors. Despite many recent advances in locoregional, interventional, and systemic therapies, the only definitive treatment for HCC is liver resection or transplantation. Unfortunately, due to poor surveillance programs, most patients with advanced HCC present at late, terminal stages, well outside the window for liver resection or transplant candidacy. This has tremendously emphasized the development of chemotherapeutic and immunotherapy alternatives. HCC is a multidisciplinary approach where interventional radiologists, radiation oncologists, surgical oncologists, and medical oncologists work together to achieve the best possible outcomes. Further understanding of tumor molecular pathways, biomarkers, and the effectiveness of combination therapy can aid in developing new treatment algorithms, which may provide a breakthrough for HCC treatment. In summary, a new diagnosis of HCC should be explored multi-disciplinary, as combining modalities from the various specialties may downstage the tumor, increase potential cure rates, and improve overall survival.
